# Educating community members in Nepal about microbial keratitis

**Published:** 2025-01-31

**Authors:** Sandip Das Sanyam

**Affiliations:** 1 Research Project Lead, Sagarmatha Choudhary Eye Hospital, Nepal.

**A poster about two farmers is encouraging agricultural workers in Nepal to seek urgent medical attention after eye injuries.**

Microbial keratitis is an infection of the cornea: the clear, dome-shaped surface that covers the front of the eye. This condition is caused by various microorganisms, including bacteria, fungi, viruses, and protozoa. It is a serious eye condition that can lead to significant pain, vision loss, and even blindness if not treated promptly and effectively.

Nepal has a high incidence of corneal abrasions and infections. Farmers and agricultural workers are at increased risk as they are exposed on a daily basis to plant material, dust and debris. If farm workers do not use protective eyewear, they are at increased risk of eye injuries that introduce microorganisms directly into the cornea. Any delays in treatment will result in the infection becoming worse, increasing the risk of sight loss. To address this, Sagarmatha Choudhary Eye Hospital (SCEH), supported by the London School of Hygiene & Tropical Medicine and the International Centre of Eye Health, UK, have developed a poster to raise awareness about corneal ulcers amongst farm workers in Siraha District, Nepal, and encourage them to seek help urgently.

The poster describes the experience of two farmers: one who sought urgent treatment, and one who delayed seeking help. Using colourful, culturally appropriate images and storytelling, the poster makes it possible for all community members, including those unable to read, to learn about the connection between their health seeking behaviour and their future health and wellbeing.

Public health researchers and the SCEH team created the poster and ensured the information was scientifically accurate. Team members undertook four rounds of revision of the poster by asking patients attending SCEH hospital in Lahan to share their understanding of the illustrations; their feedback was incorporated in subsequent versions. This helped to ensure that the posters would be widely understood.

Canvas copies of the poster were displayed in 40 health centres, and in their surrounding vilages, in the district. For a few months after the posters were put up, the team noticed an increase in the number of patients coming to the health centres with red eyes or eye injuries, with patients saying they attended because of what they learnt from the posters. However, the posters degraded over time or were removed, which meant that these gains were not sustained. A more long-term or permanent display should be considered.

See overleaf for an English translation of the poster. Editable files are available if you would like to adapt it, e.g. by using names or images better suited to your local community. Email requests to editor@cehjournal.org.

**A tale of two farmers: the story of Dukhiya and Sukhiya**

**Dukhiya (right)**, a hardworking farmer, sustained a minor injury to his eye while working in the field. Initially, he noticed redness and pain but chose to ignore it, believing it would heal on its own. As days passed, his symptoms worsened. Instead of seeking professional medical help, Dukhiya tried home remedies. Unfortunately, this delay in seeking treatment made his condition much worse. The infection spread, causing significant damage to his cornea. Eventually, he lost vision in that eye. Dukhiya's story is a stark reminder of the dangers of neglecting eye injuries and the critical need for timely medical intervention.

In contrast, **Sukhiya**, another farmer who faced a similar injury while working in the field, took immediate action. Noticing redness and pain in his eye, he promptly visited the nearby health centre in his community. The health care professionals evaluated his condition and prescribed eye drops, which Sukhiya used as directed. With timely and correct treatment, Sukhiya's eye healed quickly, and he was able to return to work without any lasting damage. Sukhiya's story highlights the importance of seeking professional medical help immediately after an eye injury to ensure a positive outcome.

**Figure:**
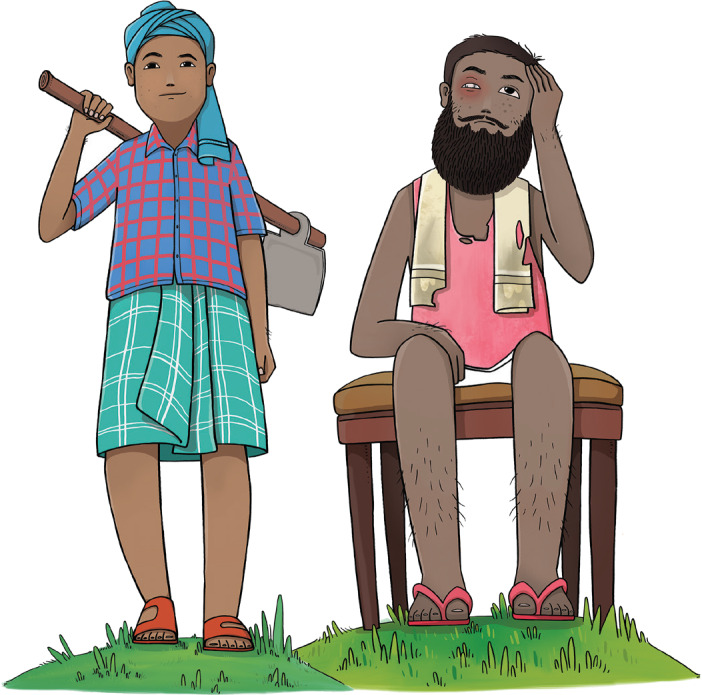
ILLUSTRATION BY SUMAN MAHARAJAN, KATHMANDU, NEPAL; CC LICENSE UNDER PREVENTION OF CORNEAL BLINDNESS COMMUNITY AWARENESS POSTER-NEPAL © 2023, SAGARMATHA CHOUDHARY EYE HOSPITAL AND LONDON SCHOOL OF HYGIENE AND TROPICAL MEDICINE CC BY-NC-SA 4.0

“The poster underscores the significance of timely medical intervention and accessible healthcare facilities in preventing severe outcomes.”

